# A co-evolutionary perspective on humans and *Mycobacterium tuberculosis* in the era of systems biology

**DOI:** 10.7554/eLife.108175

**Published:** 2026-01-07

**Authors:** Michaela T Reichmann, Liku B Tezera, Laura Denney, Hannah Schiff, Andres Vallejo, Salah Mansour, Alasdair Leslie, Diana J Garay-Baquero, Paul T Elkington

**Affiliations:** 1 NIHR Biomedical Research Centre, Clinical and Experimental Sciences Academic Unit, Faculty of Medicine, University of Southampton Southampton United Kingdom; 2 Institute for Life Sciences Southampton United Kingdom; 3 Africa Health Research Institute Durban South Africa; 4 College of Health Sciences, School of Laboratory Medicine and Medical Sciences, University of KwaZulu Natal Durban South Africa; 5 Department of Infection and Immunity, University College London London United Kingdom; Massachusetts Institute of Technology United States; University of the Witwatersrand South Africa

**Keywords:** tuberculosis, evolution, immunopathology, pulmonary, mycobacterium

## Abstract

Tuberculosis is once again the most fatal global infectious disease and has killed many more humans than any other pathogen. Despite the identification of *Mycobacterium tuberculosis* (Mtb) over 140 years ago, we have yet to control the epidemic. A central issue is the complexity of the host–pathogen interaction, with multiple underlying pathways leading to tuberculosis disease. This intricate relationship stems from the prolonged co-evolution of the pathogen with humans, resulting in diverse immunological processes leading to tuberculosis disease. Conversely, Mtb exposure may give a survival advantage through innate immune training, thereby providing selective pressure over millennia. Emerging methodologies, such as single-cell and spatial transcriptomics, offer a golden opportunity to understand the immunology unpinning this host–pathogen interaction at unprecedented resolution. However, these analyses will be fundamentally flawed if they do not consider the intricacies of human Mtb infection. Here, we propose that attempts to find single immunological mechanisms leading to tuberculosis are hindering progress, and we must embrace the complexity of multiple paths to disease to allow the systems biology era to deliver transformative solutions.

## Introduction

The study of tuberculosis (TB) pioneered infectious disease research in the modern scientific era, contributing to the formulation of Koch’s postulates demonstrating that an illness can have an infectious origin (Kaufmann, 2003). *Mycobacterium tuberculosis* (Mtb) infects and survives within macrophages, subverting the host immune response by multiple mechanisms including inhibition of phagosome maturation and downregulation of antigen presenting molecules, leading to the formation of complex immune aggregates, known as granulomas (O’Garra et al., 2013). Even though TB was the first definitively identified infection, it remains the world’s deadliest infectious disease despite well over 100 years of research (Trajman et al., 2025). This contrast raises a critical question: why is Mtb proving so resistant to human efforts to control it?

Mtb has eluded attempts to develop a fully protective vaccine, despite a partially effective vaccine being available since the 1920s. *Mycobacterium bovis* BCG was developed by sequential culture of *M. bovis* and protects children against disseminated TB but has limited protection against adult disease (Mangtani et al., 2014; Trunz et al., 2006). Although exciting progress has been made with a vaccine that reduces progression to overt TB disease by 50% when given to those with immunological evidence of latent infection in a phase II study (Tait et al., 2019), currently the immune mechanisms underpinning protection have not been identified. Subsequently, a major trial of an alternative vaccine showed no efficacy in preventing recurrence. Indeed, despite being highly immunogenic, the relapse rate tended to be higher in the vaccine group (Borges et al., 2025). Similarly, repeat BCG vaccination does not increase protection, despite inducing a strong Mtb-specific CD4 T cell response (Schmidt et al., 2025).

These difficulties highlight the priority of understanding the host–pathogen interaction more fully. We have insufficient knowledge of key steps in disease progression to develop transformative interventions. Heterogeneity across the spectrum of human TB is well described (Barry et al., 2009; Cadena et al., 2017), but the majority of fundamental investigations into disease mechanisms are based on the premise of a consistent underlying process, and that this can be understood through reductionist scientific approaches. However, clinical observations demonstrate that there are multiple paths to TB, and so seeking to define a single mechanism is likely to be flawed.

## Immunological insights from historic and recent clinical observations

Mtb has co-evolved with humans for millennia, with some estimates suggesting up to 70,000 years (Brites and Gagneux, 2015), though other analyses suggest the most recent common ancestor was ~6000 years before present (Bos et al., 2014; Kay et al., 2015). The field of paleoarchaeology provides extensive evidence of TB from the early Neolithic period in the Middle East, with approximately 5% of skeletons from a 10,000-year-old village showing signs of TB (Dutour, 2023). This was just before animal domestication and pottery, in hunter-gatherers who built stone houses, and so Mtb was already successfully transmitting in humans before the subsequent population growth that occurred with farming (Dutour, 2023). Potentially, to survive in relatively small hunter-gatherer communities, Mtb may have needed to have reduced virulence to avoid excessive deaths and a latent period to permit sustainable transmission in low population numbers (Gagneux, 2012). With the expansion and increased density of human populations, more rapid progression to TB disease can be sustained, consistent with analysis that most TB progression in high-incidence settings occurs within 1–2 years of exposure (Behr et al., 2018).

Mtb successfully persisted over the ages and then flourished in the crowded populations that occurred with the industrial revolution (Dubos and Dubos, 1987), giving rise to the modern TB era approximately 250 years ago. The fundamental cause of TB remained unknown until Koch’s seminal work (Kaufmann, 2003). Early investigations demonstrated that the first Mtb infection point was the lung base, while Mtb exits from the apices of the lungs (Ghon, 1916). This life cycle must involve several distinct host/pathogen interactions, as initially immune evasion is required for Mtb to survive, but then later immune engagement is necessary to cause the inflammation and lung destruction needed to optimize transmission (Elkington and Friedland, 2015). Cavitary lung disease leads to proliferation of extracellular bacteria and increased transmission (Yoder et al., 2004). Notably, most people (approximately 90%) initially infected with Mtb never progress to active, clinical disease (Trajman et al., 2025). In addition, in the pre-antibiotic era, the progression and regression of different lesions in the same individual were observed on chest radiographs, and one third of patients with active TB disease self-healed, showing that the host–pathogen interaction is finely balanced at all stages of infection (Dubos and Dubos, 1987).

Modern immunological techniques and the development of biologic therapies that target specific immune processes have provided extensive insight into TB disease mechanisms. The greatly increased occurrence of TB in the context of HIV co-infection, for example, highlighted immunodeficiency as a major driver of disease (Kwan and Ernst, 2011). Similarly, the occurrence of TB after anti-TNF-α therapy for autoimmune disease confirmed the importance of TNF-α in control of latent infection (Keane et al., 2001). Furthermore, genetic investigations have identified numerous immunodeficiencies via studies of Mendelian Susceptibility to Mycobacterial Diseases (MSMD), with mutations typically along the IL-12/IFN-γ/STAT signaling pathway (Jouanguy et al., 1996; Dupuis et al., 2001; Arias et al., 2024). With less clearly defined immunologic mechanisms, malnutrition is a significant risk factor for TB (Dheda et al., 2016), and food supplementation reduces TB incidence in contacts (Bhargava et al., 2023). Therefore, diverse immune deficiencies can lead to active TB.

**Figure 1.**
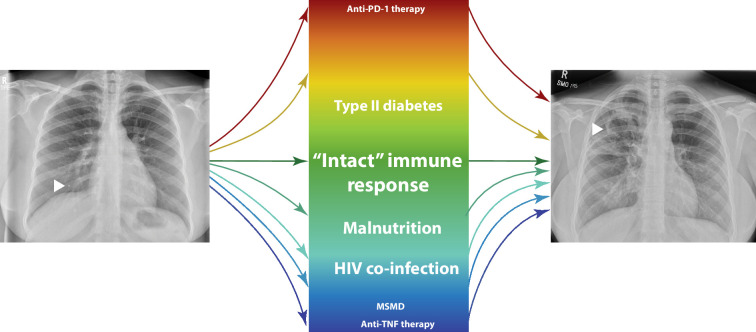
Multiple pathways can lead to tuberculosis (TB) disease from opposing immunological extremes. Generally, these can be viewed as immune deficiencies or immune excess, but the majority of patients who develop TB are relatively young with a competent immune response, illustrating the complexity of this spectrum. Though not quantitative, font size relates to contribution to global incidence. Left arrow: Ghon focus at lung base; Right arrow: cavity at lung apex.

The vast majority of patients who develop TB, however, have no clear identifiable immunodeficiency. Indeed, Comstock’s seminal study from the 1970s showed that children from Haiti with the strongest recall responses to Mtb antigens actually had the greatest subsequent risk of developing TB (Comstock et al., 1974). These observations have been replicated in more recent studies using IFN-γ release assays (IGRAs) in response to TB antigens, where higher IFN-γ production associates with increased risk of progressing to disease in both children and adults (Andrews et al., 2017; Ledesma et al., 2021). TB is most common in young adults in their immunological prime and more frequent in males than females, characterized by an excessive inflammatory response (Horton et al., 2016). The implication that TB can also be caused by immune excess is now supported by recently introduced cancer immunotherapies (Tezera et al., 2020b). Anti-PD-1 treatment, which activates the immune response and represents the immunological opposite to anti-TNF therapy, should control TB if immunodeficiency were the critical component. However, anti-PD-1 treatment can lead to rapid reactivation of latent TB infection, first identified in case reports (Elkington et al., 2018). This finding is supported by studies in mice (Lázár-Molnár et al., 2010; Barber et al., 2011), the non-human primate (Kauffman et al., 2021) and 3D cellular models (Tezera et al., 2020a), and ultimately has been validated by patient registry studies (Liu et al., 2022; Zhu et al., 2022). Similarly, type II diabetes is associated with an increased risk of TB (Dheda et al., 2016), characterized by a hyper-inflammatory immune response (Eckold et al., 2021). Therefore, diverse clinical evidence demonstrates that there are multiple immunological disturbances that can lead to TB disease (Behr et al., 2024; Figure 1).

Even when TB develops in the face of a ‘normal’ immune system, there are likely to be many different subgroups that have not yet been identified due to limitations in standard immunological profiling. A quarter of the world’s population is thought to be exposed to Mtb (Houben and Dodd, 2016), and so the vast majority of those infected with Mtb remain healthy lifelong (Trajman et al., 2025), while those who progress to active TB likely represent distinct outlier populations. The diverse causes of active TB, such as anti-TNF, MSMD, HIV, diabetes, and anti-PD1, demonstrate that patients do not reach TB by a single pathway, but instead lose the immunological balance that controls Mtb by multiple paths. These observations may explain why genetic studies have generally failed to find consistent predispositions. Evidence of heritability can be demonstrated but is often hard to validate in different populations (Schurz et al., 2024). Mutations in the macrophage endosomal protein NRAMP1, for example, were shown to associate with TB in an early seminal study (Bellamy et al., 1998), but since then, few consistent traits of genetic susceptibility to TB have been identified (Abel et al., 2014).

Recent genomic studies demonstrate the complex co-evolution of host and Mtb. The International Tuberculosis Host Genetics Consortium’s first analysis found one significant host genetic variant, human leukocyte antigen-II region (rs28383206), which conferred TB susceptibility across nine genome-wide association studies across three continents (Schurz et al., 2024). However, other variants previously associated with TB susceptibility were not replicated. Another approach using genome-to-genome analysis of paired human and Mtb samples in Peru identified another determinant on chromosome 6, rs3130660, in the flotillin-1 (FLOT1) gene (Luo et al., 2024). Together with our understanding of the co-adaptation of different Mtb lineages with human migrations (Comas et al., 2013), the host immune response to Mtb is likely to be dependent on ancestral-related genetic factors and complex host–pathogen dynamics which remain incompletely understood.

Studies of individuals who resist Mtb infection despite recurrent exposure generate a diverse list of potentially protective features, including T cell subsets or activation (Cross et al., 2024; Sun et al., 2024b; Dallmann-Sauer et al., 2025), TNF-α responses (Simmons et al., 2022), antibody activity (Lu et al., 2019), and innate immune training (Verrall et al., 2020). These human observations are supported by murine genetic experiments, which show that increased susceptibility to Mtb can result from a wide range of immunological alterations (Smith et al., 2022). Similarly, infant vaccine studies show that there are distinct patterns of response that may determine vaccine efficacy (Fletcher et al., 2016). However, despite this evidence of diversity, the majority of fundamental studies continue to seek a single underlying mechanism that leads to TB disease progression, which is incompatible with clinical observations.

Ultimately, to transmit efficiently, Mtb needs to cause pulmonary disease to then spread by airborne droplets (Yoder et al., 2004), and for Mtb, it does not matter the route taken, as long as it ends at pulmonary TB. Increasing evidence suggests that asymptomatic transmission may be important in high-incidence settings, potentially in the absence of overt pulmonary disease (Ryckman et al., 2022; Dinkele et al., 2024; Patterson et al., 2024). Experimentally, TB is a fundamentally challenging disease to model, as the interaction is prolonged and Mtb is an obligate human pathogen (Elkington et al., 2019). The primary driver of advances in immunology in the last decades has been transgenic mice (Gros and Casanova, 2023), but the mouse model of TB does not accurately reflect human disease (Young, 2009). Disease heterogeneity has been highlighted in describing clinical TB endotypes observed during active disease (DiNardo et al., 2021), with different endotypes exhibiting diverse immunological characteristics and association with outcome (DiNardo et al., 2022). However, we propose that insufficient attention has been given to the different immunological pathways that may converge on the same disease phenotype.

## Mtb’s single successful establishment in humanity

Just as new tools are providing insights into the complexity of human TB progression, advances in mycobacterial genomics are highlighting the unique nature of the human-Mtb relationship (Koleske et al., 2023). Mtb is a near-clonal organism, with evidence suggesting that there has been only one successful and sustained penetration into the human population (Comas et al., 2013; Koleske et al., 2023; Goig et al., 2025). The entire spectrum of Mtb strains globally only differs by a total of approximately 2000 SNPs (Koleske et al., 2023). This genetic conservation has persisted from the most recent common ancestor (Chiner-Oms et al., 2019) during the expansion of Mtb in human populations since the industrial revolution, which created much denser human aggregations suitable for transmission (Dubos and Dubos, 1987). This suggests that Mtb was already close to being optimized for human hosts. Further evolution may have been to increase transmission within specific populations as humans diverged genetically (Goig et al., 2025), with a recent study suggesting that Mtb may in fact be becoming attenuated to increase spread in populations (Culviner et al., 2025). Hence, Mtb may be evolving over time to optimize its transmission within humans depending on population density.

A very similar organism, *M. canetti*, can cause disease but cannot transmit from human to human (Yenew et al., 2023). Similarly, *M. bovis* is 99.9% identical to Mtb (Garnier et al., 2003), but has never achieved sustained human-to-human transmission, despite many millions of human exposures during the pre-pasteurization era and common lymph node infections (Goig et al., 2025). Therefore, human TB is caused exclusively by Mtb, unlike other very closely related mycobacteria, which maintain infection cycles in other higher mammals but not humans (Goig et al., 2025). Clues to the key pathogenic mechanisms may lie in the differences between mycobacterial species (Danchuk et al., 2025), but ultimately, similarities between Mtb strains must be critical to their ongoing success. For example, the hyper-conservation of T cell epitopes may be evidence that Mtb manipulates the host immune response to favor disease and transmission (Comas et al., 2010). Given the size of the Mtb genome, identifying the critical conserved features will be challenging, especially as half of its genes still lack a known function 25 years after it was first sequenced (Nathan, 2023).

One highly intriguing proposition is that latent TB may itself give humans an evolutionary advantage (Nathan, 2023). Exposure to Mtb modifies innate immune training via reprogramming hematopoietic stem cells (Khan et al., 2020), and so a mechanism whereby Mtb could protect from other fatal infections is plausible. Similarly, *M. bovis* BCG, a live-attenuated strain of *M. bovis*, causes innate immune training (Kaufmann et al., 2018), suggesting this effect is common to Mtb and BCG. BCG vaccination reduces mortality much more significantly than can be explained by the effect on TB incidence alone (Jurczak and Druszczynska, 2025), with a protective effect confirmed in diverse studies (Moorlag et al., 2019). For example, BCG reduces viral infections in infants in Africa (Stensballe et al., 2005) and experimentally challenged adults (Arts et al., 2018), and associates with improved survival in Europe (Rieckmann et al., 2017). Mtb and BCG can protect against SARS-CoV2 infection (Rosas Mejia et al., 2022; Hilligan et al., 2022), although this did not translate to efficacy in a clinical trial (Pittet et al., 2023). Together, these observations strongly imply that mycobacterial infection protects from other infectious causes of death.

Mtb almost certainly first became established in humans in East Africa (Comas et al., 2013; Goig et al., 2025), potentially about 70,000 years ago (Brites and Gagneux, 2015), although this timeline is debated. Several human migrations out of Africa are thought to have preceded this date, but all ultimately became extinct, with the first sustained human dispersal 60–70,000 years ago (Vallini et al., 2024). Mtb diversity mirrors human mitochondrial genome diversity, further implying that Mtb disseminated with human populations from East Africa (Comas et al., 2013). The primary selective pressure in these early communities would have been infectious disease (Dobson and Carper, 1996). This raises a novel hypothesis that a survival advantage for the first successful human migrants out of Africa was Mtb circulating in the community, reducing mortality from other infectious diseases and thereby enabling sustainable population growth.

**Figure 2.**
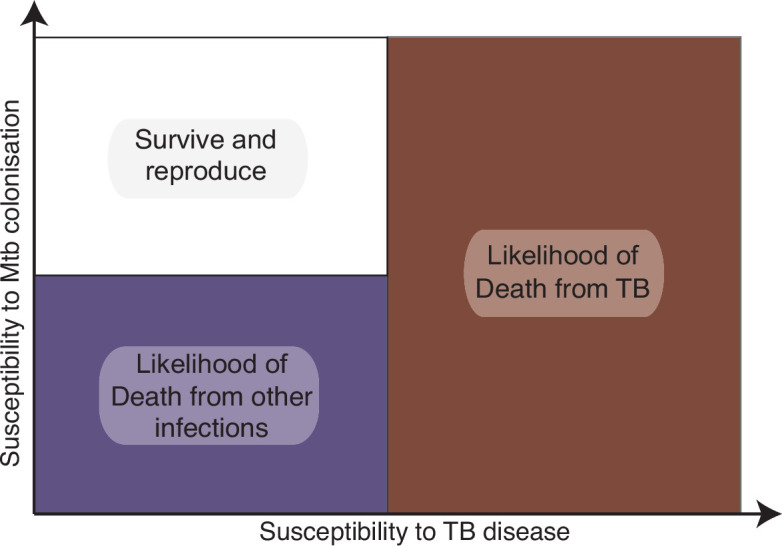
Selection pressure of prolonged co-evolution favors individuals permissive to asymptomatic *Mycobacterium tuberculosis* (Mtb) colonization but resistant to active disease. Over millennia, Mtb circulation in society will remove genetic traits that cause high susceptibility to active tuberculosis (TB) infection. Perhaps less intuitively, if Mtb generates trained immunity that protects against other fatal diseases, individuals with low susceptibility to initial Mtb infection will also be selected against due to increased mortality from other infections. The resulting population would then reflect modern humans: highly susceptible to initial Mtb colonization but with low susceptibility to TB disease. The figure illustrates the selective pressure concept, the increase in risk is not binary but gradual, with susceptibility determined by multiple aspects of the host immune response.

Mtb transmission may have benefitted humans by increasing innate immune resistance to infection at the cost of 10% disease penetrance that permits Mtb propagation. This selective pressure over many millennia would progressively remove genotypes that lead to high susceptibility to TB, but equally would select against individuals with complete resistance to initial Mtb infection (Figure 2). This could explain why consistent genetic traits for susceptibility or resistance to TB have been hard to identify (Abel et al., 2014). More recent mass infection events, such as the smallpox epidemics that killed approximately 25% of the population (Dobson and Carper, 1996), may have further favored individuals immunologically trained by Mtb. Likewise, successive waves of plague killed approximately 25% of the population, at the end of the Roman empire and then in the European middle ages, adding to selective pressure from endemic infections (Little, 2007; Benedictow, 2004). If humans have been selected to be permissive to Mtb infection but resistant to TB disease, which could be regarded as colonization, it suggests the development of active disease must be a relatively unusual event in a subset of outlier individuals. In some sense, we could be regarded as having a symbiotic relationship with Mtb, with disease representing a necessary evil, caused by an imbalance in the predominantly stable host–pathogen interaction (Divangahi et al., 2018; Olive and Sassetti, 2018).

## The host–pathogen interaction at a cellular level

The early histological era described the wide range of human TB lesions and granuloma types and identified TB as a disease characterized by spatial organization (Hunter, 2016). Classically, the granuloma has been proposed to be where the outcome of the host–pathogen interaction is determined (Pagán and Ramakrishnan, 2018). Recent methodological advances are permitting much greater dissection of events and further highlight the importance of spatial organization within the granuloma (Sawyer et al., 2023; McCaffrey et al., 2022; Marakalala et al., 2016). However, just as the early X-ray era showed some lesions progressing and some regressing, these studies demonstrate the great heterogeneity between granuloma types. Studies in the non-human primate have allowed investigation into features of progressing and controlling granulomas, identifying potential correlates of immune control (Gideon et al., 2022), but even this model only partially recapitulates human disease.

Furthermore, the recent spatial studies highlight the complexity of cellular players, including the established fulcrum of macrophages and T cells, but additionally the importance of B cells, neutrophils, and fibroblasts. For example, fibroblasts are emerging as important immune regulators in other lung diseases (Ghonim et al., 2023), and so it seems highly likely that they play an active role in TB-related inflammation. Fibroblast zonation can lead to feed-forward inflammatory loops and so may propagate disease (Davidson et al., 2021). Consequently, the full spectrum of cell types in Mtb-infected lesions needs to be considered. Given that multiple underlying immunological pathways can lead to active TB, it seems unlikely that a single cellular component will fully explain the balance between Mtb containment and progression to active disease. However, the majority of studies continue to look for a single consistent immune mechanism; discussions rarely state ‘this is one of several potential routes to TB disease’ (Sun et al., 2024b; Gideon et al., 2022; Winchell et al., 2023; Swanson et al., 2023; Proulx et al., 2025).

**Figure 3.**
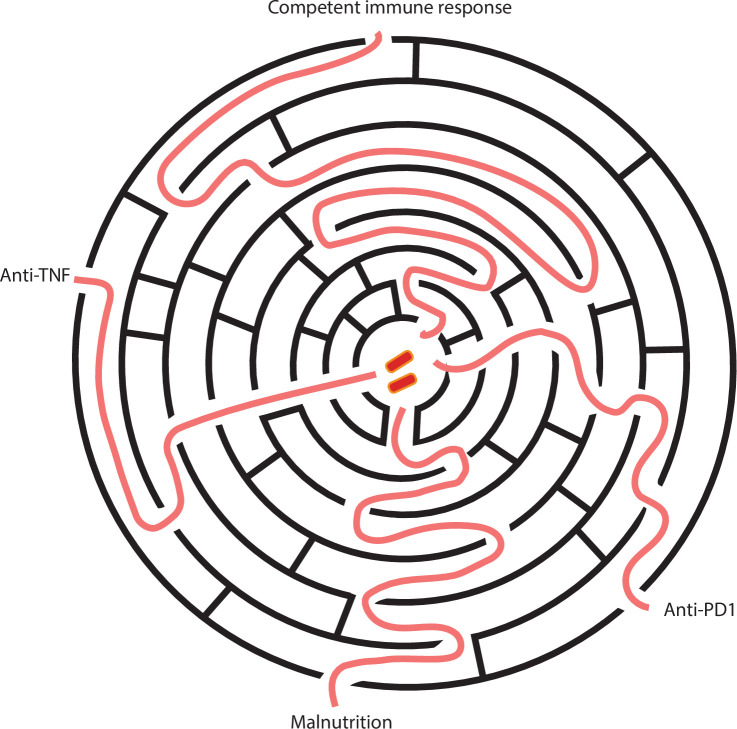
Schematic of the interactions needed for *Mycobacterium tuberculosis* (Mtb) to escape the host immune response. As control of Mtb requires a coordinated host response, there are multiple sequences of immune events that can ultimately result in progression to active tuberculosis (TB) disease. A major immune disturbance, such as TNF-α or PD-1 inhibition, gives a relatively direct pathway to active TB. However, most individuals develop TB due to a series of less apparent immune events and no clear global immune disturbance that can be identified by current immune profiling approaches.

Considering the clinical and experimental evidence, immunological failure is likely to be a multistep process, whereby either one large deficit or numerous small imbalances can lead to progression and disease (Figure 3). Mtb and humans interact over many years, as the pathogen is difficult to eradicate due to its highly evolved survival mechanisms (Russell, 2011), and therefore in those individuals in whom it survives, there is a long period where it can escape host control. Potentially, these different paths may ultimately cross or converge; if so, understanding the key nodes will allow more broadly effective treatments to emerge. For example, lung extracellular matrix degradation could be regarded as a final common pathway (Elkington et al., 2022), but, again, this may result from different collagenases including macrophage-derived MMP-1 or neutrophil-derived MMP-8 (Elkington et al., 2011; Ong et al., 2015).

## Emerging methodologies and the challenge of data analysis

The recent adoption of ‘omic’ methodologies, including single-cell transcriptomics, spatial transcriptomics, and proteomics, offers unprecedented opportunities to dissect the mechanisms of human TB pathogenesis and accelerate the development of effective interventions. These approaches generate large-scale, complex datasets that capture the heterogeneity of TB lesions. However, this complexity also presents significant analytical challenges. Standard computational pipelines, if not adapted to account for the biological and technical variability in these data, are unlikely to deliver robust or reproducible insights. A major obstacle is the integration of data from multiple studies and platforms, which can differ both within a single omic layer (horizontal integration) and across multiple omic layers (vertical integration) (Zheng et al., 2024). Such integration risks data loss and inconsistent results, especially when data are not harmonized.

The prolonged co-evolution between host and pathogen has resulted in multiple immunological pathways to active TB, significantly adding to the biological heterogeneity that complicates the analysis and interpretation of multi-omic data. Addressing this complexity requires well-annotated clinical cohorts that capture the full spectrum of TB heterogeneity, ideally with longitudinal outcome data rather than single time-point snapshots. Comprehensive clinical descriptors and associated data such as laboratory analyses and chest X-rays will permit definition of each clinical phenotype. To accommodate the diversity of disease pathways, novel bioinformatic approaches are needed that move beyond the assumption of a single sequence of events that results in active TB.

The power of multi-omic approaches to reveal complex molecular relationships depends on both the quality of the omic data and the fit between experimental design and data integration strategies. For example, correlation-based integration requires matched samples across omics, sufficient sample numbers, and comparable variance structures. These requirements are often overlooked, leading to insufficient power, noisy data, and unrealistic integration (Tarazona et al., 2020). Each omic technology brings its own challenges. Signal-to-noise ratios differ across modalities, and appropriate algorithms are needed to estimate sample size and power for each. Notably, using the same sample numbers across modalities does not ensure comparable statistical power; achieving equal power can require unbalanced sample sizes (Tarazona et al., 2021). Other recognized challenges include the interpretation and validation of multi-omic models, standardized annotation, and data sharing (Tarazona et al., 2021). Recent developments include web-based, user-friendly tools that enable both knowledge- and data-driven multi-omic integration (Ewald et al., 2024). Importantly, a wider use of artificial intelligence (AI) is transforming omics data analysis by providing robust methods to interpret complex biological datasets (Ahmed et al., 2024). Machine learning and deep learning techniques are now routinely applied to DNA, transcriptomic, proteomic, and metabolomic data, enabling more integrated and comprehensive analyses (Ahmed et al., 2024). In proteomics, for example, AI-driven approaches have enhanced peptide measurement predictions and accelerated biomarker discovery, often outperforming conventional assays (Mann et al., 2021). To improve interpretability, explainable AI (XAI) methods are increasingly employed, with feature relevance mapping and visual explanations emerging as preferred post hoc strategies (Toussaint et al., 2023). However, despite these developments, significant challenges remain in implementing XAI. Further research is needed to overcome these barriers and unlock the full translational potential of AI in omics analysis (Mann et al., 2021; Toussaint et al., 2023). Ultimately, the utility of these innovations will depend on the functional validation of the resulting models.

Given the complexity of human TB, careful study design and unbiased integration methods that accommodate data limitations are essential. Spatial context is particularly important, as TB pathology involves three-dimensional immune responses. Extracellular matrix remodeling is a hallmark of TB granulomas (Elkington et al., 2022), and the matrix regulates host cell biology (Bansaccal et al., 2023). Consequently, multiple data inputs such as matrix composition and organization may be needed alongside host and Mtb transcriptomic data. Ultimately, local cellular events must be modeled at the tissue level, providing a second layer of computational complexity (Palla et al., 2022).

Addressing these challenges will require substantial investment in analytical capacity. Multi-omics is already transforming our understanding of disease heterogeneity, facilitating the identification of previously unrecognized subgroups, refining prognostic and therapeutic approaches, and providing deeper mechanistic insights. This strategy has successfully stratified rare tumors (Sun et al., 2024a), profiled healthy populations (Halama et al., 2024), and enabled health screening to reveal previously hidden disease and risk subgroups (Garg et al., 2024), supporting the shift toward precision medicine. As human disease processes are rarely uniform, advances in multi-omic study design and analysis for TB will likely benefit a broad range of conditions. Ultimately, this comprehensive understanding of disease pathophysiology can then lead to more targeted and stratified treatment, although implementation will require developments in companion diagnostics to accurately stratify patients.

## Implications of diverse disease pathways for new TB interventions

Ultimately, the complexity of human TB underlies our inability to deploy a transformative intervention, and so global mortality remains depressingly high. The human–Mtb interaction is so closely co-evolved that experimental findings need to be interpreted in light of human disease phenomena. Multiomic studies in TB are currently being undertaken on small numbers due to cost and challenges in obtaining appropriate clinical samples, in specific regions, and so are unlikely to reflect the global heterogeneity. Therefore, results need to be interpreted with caution and wider studies will be needed to confirm the generalizability of findings. A critical aspect will be carefully curated and fully accessible metadata, so that as the body of omic datasets on human TB increases, they can be accurately integrated into wider analyses. Recurrent mining of these datasets is likely to be fundamental to understanding the breadth of TB pathogenesis. Missing metadata makes interpretation difficult, and in worst cases, misleading. In addition, innovative computational approaches will be required whereby the analysis specifically accommodates multiple immune pathways to disease.

Given the toll that TB takes in the poorest parts of the world, we have a moral imperative to end the epidemic (Reid et al., 2023). To achieve this, we must acknowledge the complexity of human TB that has resulted from our prolonged co-evolution with the pathogen and the selective pressure of persistent Mtb exposure over millennia. Success depends on integrating the full spectrum of TB disease into our bioinformatic analyses, and ultimately understanding TB’s complexity can then inform logical interventions. If we seek a single mechanistic explanation of TB disease, this seems to be unlikely to be successful.

## References

[bib1] Abel L, El-Baghdadi J, Bousfiha AA, Casanova JL, Schurr E (2014). Human genetics of tuberculosis: a long and winding road. Philosophical Transactions of the Royal Society of London. Series B, Biological Sciences.

[bib2] Ahmed Z, Wan S, Zhang F, Zhong W (2024). Artificial intelligence for omics data analysis. BMC Methods.

[bib3] Andrews JR, Nemes E, Tameris M, Landry BS, Mahomed H, McClain JB, Fletcher HA, Hanekom WA, Wood R, McShane H, Scriba TJ, Hatherill M (2017). Serial QuantiFERON testing and tuberculosis disease risk among young children: an observational cohort study. The Lancet. Respiratory Medicine.

[bib4] Arias AA, Neehus A-L, Ogishi M, Meynier V, Krebs A, Lazarov T, Lee AM, Arango-Franco CA, Yang R, Orrego J, Corcini Berndt M, Rojas J, Li H, Rinchai D, Erazo-Borrás L, Han JE, Pillay B, Ponsin K, Chaldebas M, Philippot Q, Bohlen J, Rosain J, Le Voyer T, Janotte T, Amarajeeva K, Soudée C, Brollo M, Wiegmann K, Marquant Q, Seeleuthner Y, Lee D, Lainé C, Kloos D, Bailey R, Bastard P, Keating N, Rapaport F, Khan T, Moncada-Vélez M, Carmona MC, Obando C, Alvarez J, Cataño JC, Martínez-Rosado LL, Sanchez JP, Tejada-Giraldo M, L’Honneur A-S, Agudelo ML, Perez-Zapata LJ, Arboleda DM, Alzate JF, Cabarcas F, Zuluaga A, Pelham SJ, Ensser A, Schmidt M, Velásquez-Lopera MM, Jouanguy E, Puel A, Krönke M, Ghirardello S, Borghesi A, Pahari S, Boisson B, Pittaluga S, Ma CS, Emile J-F, Notarangelo LD, Tangye SG, Marr N, Lachmann N, Salvator H, Schlesinger LS, Zhang P, Glickman MS, Nathan CF, Geissmann F, Abel L, Franco JL, Bustamante J, Casanova J-L, Boisson-Dupuis S (2024). Tuberculosis in otherwise healthy adults with inherited TNF deficiency. Nature.

[bib5] Arts RJW, Moorlag S, Novakovic B, Li Y, Wang SY, Oosting M, Kumar V, Xavier RJ, Wijmenga C, Joosten LAB, Reusken C, Benn CS, Aaby P, Koopmans MP, Stunnenberg HG, van Crevel R, Netea MG (2018). BCG vaccination protects against experimental viral infection in humans through the induction of cytokines associated with trained immunity. Cell Host & Microbe.

[bib6] Bansaccal N, Vieugue P, Sarate R, Song Y, Minguijon E, Miroshnikova YA, Zeuschner D, Collin A, Allard J, Engelman D, Delaunois AL, Liagre M, de Groote L, Timmerman E, Van Haver D, Impens F, Salmon I, Wickström SA, Sifrim A, Blanpain C (2023). The extracellular matrix dictates regional competence for tumour initiation. Nature.

[bib7] Barber DL, Mayer-Barber KD, Feng CG, Sharpe AH, Sher A (2011). CD4 T cells promote rather than control tuberculosis in the absence of PD-1-mediated inhibition. Journal of Immunology.

[bib8] Barry CE, Boshoff HI, Dartois V, Dick T, Ehrt S, Flynn J, Schnappinger D, Wilkinson RJ, Young D (2009). The spectrum of latent tuberculosis: rethinking the biology and intervention strategies. Nature Reviews. Microbiology.

[bib9] Behr MA, Edelstein PH, Ramakrishnan L (2018). Revisiting the timetable of tuberculosis. BMJ.

[bib10] Behr MA, Edelstein PH, Ramakrishnan L (2024). Rethinking the burden of latent tuberculosis to reprioritize research. Nature Microbiology.

[bib11] Bellamy R, Ruwende C, Corrah T, McAdam KP, Whittle HC, Hill AV (1998). Variations in the NRAMP1 gene and susceptibility to tuberculosis in West Africans. The New England Journal of Medicine.

[bib12] Benedictow OJ (2004). The Black Death, 1346-1353: The Complete History.

[bib13] Bhargava A, Bhargava M, Meher A, Benedetti A, Velayutham B, Sai Teja G, Watson B, Barik G, Pathak RR, Prasad R, Dayal R, Madhukeshwar AK, Chadha V, Pai M, Joshi R, Menzies D, Swaminathan S (2023). Nutritional supplementation to prevent tuberculosis incidence in household contacts of patients with pulmonary tuberculosis in India (RATIONS): a field-based, open-label, cluster-randomised, controlled trial. Lancet.

[bib14] Borges ÁH, Russell M, Tait D, Scriba TJ, Nemes E, Skallerup P, van Brakel E, Cabibbe AM, Cirillo DM, Leuvennink-Steyn M, Rutkowski KT, Wood GK, Thierry-Carstensen B, Tingskov PN, Meldgaard EC, Kristiansen MP, Søndergaard RE, Hansen CH, Follmann F, Jensen CG, Gela A, Ntinginya NE, Ruhwald M, Shenje J, White L, Innes C, Selepe P, Ngaraguza B, Holmgren C, Collings T, Andersen P, Dawson R, Churchyard G, Sabi I, Diacon AH, Mortensen R, Hatherill M, POR TB study group (2025). Immunogenicity, safety, and efficacy of the vaccine H56:IC31 in reducing the rate of tuberculosis disease recurrence in HIV-negative adults successfully treated for drug-susceptible pulmonary tuberculosis: a double-blind, randomised, placebo-controlled, phase 2b trial. The Lancet. Infectious Diseases.

[bib15] Bos KI, Harkins KM, Herbig A, Coscolla M, Weber N, Comas I, Forrest SA, Bryant JM, Harris SR, Schuenemann VJ, Campbell TJ, Majander K, Wilbur AK, Guichon RA, Wolfe Steadman DL, Cook DC, Niemann S, Behr MA, Zumarraga M, Bastida R, Huson D, Nieselt K, Young D, Parkhill J, Buikstra JE, Gagneux S, Stone AC, Krause J (2014). Pre-Columbian mycobacterial genomes reveal seals as a source of New World human tuberculosis. Nature.

[bib16] Brites D, Gagneux S (2015). Co-evolution of *Mycobacterium tuberculosis* and *Homo sapiens*. Immunological Reviews.

[bib17] Cadena AM, Fortune SM, Flynn JL (2017). Heterogeneity in tuberculosis. Nature Reviews. Immunology.

[bib18] Chiner-Oms Á, Sánchez-Busó L, Corander J, Gagneux S, Harris SR, Young D, González-Candelas F, Comas I (2019). Genomic determinants of speciation and spread of the *Mycobacterium tuberculosis* complex. Science Advances.

[bib19] Comas I, Chakravartti J, Small PM, Galagan J, Niemann S, Kremer K, Ernst JD, Gagneux S (2010). Human T cell epitopes of *Mycobacterium tuberculosis* are evolutionarily hyperconserved. Nature Genetics.

[bib20] Comas I, Coscolla M, Luo T, Borrell S, Holt KE, Kato-Maeda M, Parkhill J, Malla B, Berg S, Thwaites G, Yeboah-Manu D, Bothamley G, Mei J, Wei L, Bentley S, Harris SR, Niemann S, Diel R, Aseffa A, Gao Q, Young D, Gagneux S (2013). Out-of-Africa migration and Neolithic coexpansion of *Mycobacterium tuberculosis* with modern humans. Nature Genetics.

[bib21] Comstock GW, Livesay VT, Woolpert SF (1974). The prognosis of a positive tuberculin reaction in childhood and adolescence. American Journal of Epidemiology.

[bib22] Cross DL, Layton ED, Yu KK, Smith MT, Aguilar MS, Li S, Wilcox EC, Chapuis AG, Mayanja-Kizza H, Stein CM, Boom WH, Hawn TR, Bradley P, Newell EW, Seshadri C (2024). MR1-restricted T cell clonotypes are associated with “resistance” to *Mycobacterium tuberculosis* infection. JCI Insight.

[bib23] Culviner PH, Frey AM, Liu Q, Ha DTM, Thai PVK, Thu DDA, Quang NL, Calderon R, Lecca L, Caws M, Dunstan SJ, Murray MB, Thuong NTT, Fortune SM (2025). Evolution of *Mycobacterium tuberculosis* transcription regulation is associated with increased transmission and drug resistance. Cell.

[bib24] Dallmann-Sauer M, Fava VM, Malherbe ST, MacDonald CE, Orlova M, Kroon EE, Cobat A, Boisson-Dupuis S, Hoal EG, Abel L, Möller M, Casanova JL, Walzl G, Du Plessis N, Schurr E (2025). *Mycobacterium tuberculosis* resisters despite HIV exhibit activated T cells and macrophages in their pulmonary alveoli. The Journal of Clinical Investigation.

[bib25] Danchuk SN, Duffy SC, Sullivan J, Beenish Rufai S, McIntosh FA, Lupien A, Harrison LB, Ghasemi Goojani H, Taylor L, Wei Y, Joubert P, Mortensen R, Chen JM, Niroula N, Stevens R, Norleen C, Kapur V, Behr MA (2025). Virulence hierarchies within the *Mycobacterium tuberculosis* complex. PNAS.

[bib26] Davidson S, Coles M, Thomas T, Kollias G, Ludewig B, Turley S, Brenner M, Buckley CD (2021). Fibroblasts as immune regulators in infection, inflammation and cancer. Nature Reviews. Immunology.

[bib27] Dheda K, Barry CE, Maartens G (2016). Tuberculosis. Lancet.

[bib28] DiNardo AR, Nishiguchi T, Grimm SL, Schlesinger LS, Graviss EA, Cirillo JD, Coarfa C, Mandalakas AM, Heyckendorf J, Kaufmann SHE, Lange C, Netea MG, Van Crevel R (2021). Tuberculosis endotypes to guide stratified host-directed therapy. Med.

[bib29] DiNardo AR, Gandhi T, Heyckendorf J, Grimm SL, Rajapakshe K, Nishiguchi T, Reimann M, Kirchner HL, Kahari J, Dlamini Q, Lange C, Goldmann T, Marwitz S, Cirillo JD, Kaufmann SHE, Netea MG, van Crevel R, Mandalakas AM, Coarfa C, DZIF-TB cohort study group (2022). Gene expression signatures identify biologically and clinically distinct tuberculosis endotypes. The European Respiratory Journal.

[bib30] Dinkele R, Gessner S, Patterson B, McKerry A, Hoosen Z, Vazi A, Seldon R, Koch A, Warner DF, Wood R (2024). Persistent *Mycobacterium tuberculosis* bioaerosol release in a tuberculosis-endemic setting. iScience.

[bib31] Divangahi M, Khan N, Kaufmann E (2018). Beyond killing *Mycobacterium tuberculosis*: disease tolerance. Frontiers in Immunology.

[bib32] Dobson AP, Carper ER (1996). Infectious diseases and human population history. BioScience.

[bib33] Dubos R, Dubos J (1987). The White Plague: Tuberculosis, Man, and Society.

[bib34] Dupuis S, Dargemont C, Fieschi C, Thomassin N, Rosenzweig S, Harris J, Holland SM, Schreiber RD, Casanova JL (2001). Impairment of mycobacterial but not viral immunity by a germline human STAT1 mutation. Science.

[bib35] Dutour O (2023). The paleopathology and paleoepidemiology of Upper paleolithic tuberculosis: review of evidence and hypotheses. Tuberculosis.

[bib36] Eckold C, Kumar V, Weiner J, Alisjahbana B, Riza A-L, Ronacher K, Coronel J, Kerry-Barnard S, Malherbe ST, Kleynhans L, Stanley K, Ruslami R, Ioana M, Ugarte-Gil C, Walzl G, van Crevel R, Wijmenga C, Critchley JA, Dockrell HM, Cliff JM, TANDEM consortium (2021). Impact of intermediate hyperglycemia and diabetes on immune dysfunction in tuberculosis. Clinical Infectious Diseases.

[bib37] Elkington Paul, Shiomi T, Breen R, Nuttall RK, Ugarte-Gil CA, Walker NF, Saraiva L, Pedersen B, Mauri F, Lipman M, Edwards DR, Robertson BD, D’Armiento J, Friedland JS (2011). MMP-1 drives immunopathology in human tuberculosis and transgenic mice. The Journal of Clinical Investigation.

[bib38] Elkington PT, Friedland JS (2015). Permutations of time and place in tuberculosis. The Lancet. Infectious Diseases.

[bib39] Elkington PT, Bateman AC, Thomas GJ, Ottensmeier CH (2018). Implications of tuberculosis reactivation after immune checkpoint inhibition. American Journal of Respiratory and Critical Care Medicine.

[bib40] Elkington P, Lerm M, Kapoor N, Mahon R, Pienaar E, Huh D, Kaushal D, Schlesinger LS (2019). In vitro granuloma models of tuberculosis: potential and challenges. The Journal of Infectious Diseases.

[bib41] Elkington P, Polak ME, Reichmann MT, Leslie A (2022). Understanding the tuberculosis granuloma: the matrix revolutions. Trends in Molecular Medicine.

[bib42] Ewald JD, Zhou G, Lu Y, Kolic J, Ellis C, Johnson JD, Macdonald PE, Xia J (2024). Web-based multi-omics integration using the Analyst software suite. Nature Protocols.

[bib43] Fletcher HA, Filali-Mouhim A, Nemes E, Hawkridge A, Keyser A, Njikan S, Hatherill M, Scriba TJ, Abel B, Kagina BM, Veldsman A, Agudelo NM, Kaplan G, Hussey GD, Sekaly R-P, Hanekom WA, BCG study team (2016). Human newborn bacille Calmette-Guérin vaccination and risk of tuberculosis disease: a case-control study. BMC Medicine.

[bib44] Gagneux S (2012). Host-pathogen coevolution in human tuberculosis. Philosophical Transactions of the Royal Society of London. Series B, Biological Sciences.

[bib45] Garg M, Karpinski M, Matelska D, Middleton L, Burren OS, Hu F, Wheeler E, Smith KR, Fabre MA, Mitchell J, O’Neill A, Ashley EA, Harper AR, Wang Q, Dhindsa RS, Petrovski S, Vitsios D (2024). Disease prediction with multi-omics and biomarkers empowers case-control genetic discoveries in the UK Biobank. Nature Genetics.

[bib46] Garnier T, Eiglmeier K, Camus J-C, Medina N, Mansoor H, Pryor M, Duthoy S, Grondin S, Lacroix C, Monsempe C, Simon S, Harris B, Atkin R, Doggett J, Mayes R, Keating L, Wheeler PR, Parkhill J, Barrell BG, Cole ST, Gordon SV, Hewinson RG (2003). The complete genome sequence of *Mycobacterium bovis*. PNAS.

[bib47] Ghon A (1916). The Primary Lung Focus of Tuberculosis in Children.

[bib48] Ghonim MA, Boyd DF, Flerlage T, Thomas PG (2023). Pulmonary inflammation and fibroblast immunoregulation: from bench to bedside. The Journal of Clinical Investigation.

[bib49] Gideon HP, Hughes TK, Tzouanas CN, Wadsworth MH, Tu AA, Gierahn TM, Peters JM, Hopkins FF, Wei JR, Kummerlowe C, Grant NL, Nargan K, Phuah JY, Borish HJ, Maiello P, White AG, Winchell CG, Nyquist SK, Ganchua SKC, Myers A, Patel KV, Ameel CL, Cochran CT, Ibrahim S, Tomko JA, Frye LJ, Rosenberg JM, Shih A, Chao M, Klein E, Scanga CA, Ordovas-Montanes J, Berger B, Mattila JT, Madansein R, Love JC, Lin PL, Leslie A, Behar SM, Bryson B, Flynn JL, Fortune SM, Shalek AK (2022). Multimodal profiling of lung granulomas in macaques reveals cellular correlates of tuberculosis control. Immunity.

[bib50] Goig GA, Windels EM, Loiseau C, Stritt C, Biru L, Borrell S, Brites D, Gagneux S (2025). Ecology, global diversity and evolutionary mechanisms in the *Mycobacterium tuberculosis* complex. Nature Reviews. Microbiology.

[bib51] Gros P, Casanova JL (2023). Reconciling mouse and human immunology at the altar of genetics. Annual Review of Immunology.

[bib52] Halama A, Zaghlool S, Thareja G, Kader S, Al Muftah W, Mook-Kanamori M, Sarwath H, Mohamoud YA, Stephan N, Ameling S, Pucic Baković M, Krumsiek J, Prehn C, Adamski J, Schwenk JM, Friedrich N, Völker U, Wuhrer M, Lauc G, Najafi-Shoushtari SH, Malek JA, Graumann J, Mook-Kanamori D, Schmidt F, Suhre K (2024). A roadmap to the molecular human linking multiomics with population traits and diabetes subtypes. Nature Communications.

[bib53] Hilligan KL, Namasivayam S, Clancy CS, O’Mard D, Oland SD, Robertson SJ, Baker PJ, Castro E, Garza NL, Lafont BAP, Johnson R, Ronchese F, Mayer-Barber KD, Best SM, Sher A (2022). Intravenous administration of BCG protects mice against lethal SARS-CoV-2 challenge. The Journal of Experimental Medicine.

[bib54] Horton KC, MacPherson P, Houben RMGJ, White RG, Corbett EL (2016). Sex differences in tuberculosis burden and notifications in low- and middle-income countries: a systematic review and meta-analysis. PLOS Medicine.

[bib55] Houben R, Dodd PJ (2016). The global burden of latent tuberculosis infection: a re-estimation using mathematical modelling. PLOS Medicine.

[bib56] Hunter RL (2016). Tuberculosis as a three-act play: a new paradigm for the pathogenesis of pulmonary tuberculosis. Tuberculosis.

[bib57] Jouanguy E, Altare F, Lamhamedi S, Revy P, Emile JF, Newport M, Levin M, Blanche S, Seboun E, Fischer A, Casanova JL (1996). Interferon-gamma-receptor deficiency in an infant with fatal bacille Calmette-Guérin infection. The New England Journal of Medicine.

[bib58] Jurczak M, Druszczynska M (2025). Beyond tuberculosis: the surprising immunological benefits of the Bacillus Calmette-Guérin (BCG) vaccine in infectious, auto-immune, and inflammatory diseases. Pathogens.

[bib59] Kauffman KD, Sakai S, Lora NE, Namasivayam S, Baker PJ, Kamenyeva O, Foreman TW, Nelson CE, Oliveira-de-Souza D, Vinhaes CL, Yaniv Z, Lindestam Arleham CS, Sette A, Freeman GJ, Moore R, Sher A, Mayer-Barber KD, Andrade BB, Kabat J, Via LE, Barber DL, NIAID/DIR Tuberculosis Imaging Program (2021). PD-1 blockade exacerbates *Mycobacterium tuberculosis* infection in rhesus macaques. Science Immunology.

[bib60] Kaufmann SHE (2003). A short history of Robert Koch’s fight against tuberculosis: those who do not remember the past are condemned to repeat it. Tuberculosis.

[bib61] Kaufmann E, Sanz J, Dunn JL, Khan N, Mendonça LE, Pacis A, Tzelepis F, Pernet E, Dumaine A, Grenier JC, Mailhot-Léonard F, Ahmed E, Belle J, Besla R, Mazer B, King IL, Nijnik A, Robbins CS, Barreiro LB, Divangahi M (2018). BCG educates hematopoietic stem cells to generate protective innate immunity against tuberculosis. Cell.

[bib62] Kay GL, Sergeant MJ, Zhou Z, Chan JZ-M, Millard A, Quick J, Szikossy I, Pap I, Spigelman M, Loman NJ, Achtman M, Donoghue HD, Pallen MJ (2015). Eighteenth-century genomes show that mixed infections were common at time of peak tuberculosis in Europe. Nature Communications.

[bib63] Keane J, Gershon S, Wise RP, Mirabile-Levens E, Kasznica J, Schwieterman WD, Siegel JN, Braun MM (2001). Tuberculosis associated with infliximab, a tumor necrosis factor alpha-neutralizing agent. The New England Journal of Medicine.

[bib64] Khan N, Downey J, Sanz J, Kaufmann E, Blankenhaus B, Pacis A, Pernet E, Ahmed E, Cardoso S, Nijnik A, Mazer B, Sassetti C, Behr MA, Soares MP, Barreiro LB, Divangahi M (2020). *M. tuberculosis* reprograms hematopoietic stem cells to limit myelopoiesis and impair trained immunity. Cell.

[bib65] Koleske BN, Jacobs WR, Bishai WR (2023). The *Mycobacterium tuberculosis* genome at 25 years: lessons and lingering questions. The Journal of Clinical Investigation.

[bib66] Kwan CK, Ernst JD (2011). HIV and tuberculosis: a deadly human syndemic. Clinical Microbiology Reviews.

[bib67] Lázár-Molnár E, Chen B, Sweeney KA, Wang EJ, Liu W, Lin J, Porcelli SA, Almo SC, Nathenson SG, Jacobs WR (2010). Programmed death-1 (PD-1)-deficient mice are extraordinarily sensitive to tuberculosis. PNAS.

[bib68] Ledesma JR, Ma J, Zheng P, Ross JM, Vos T, Kyu HH (2021). Interferon-gamma release assay levels and risk of progression to active tuberculosis: a systematic review and dose-response meta-regression analysis. BMC Infectious Diseases.

[bib69] Little LK (2007). Plague and the End of Antiquity: The Pandemic of 541-750.

[bib70] Liu K, Wang D, Yao C, Qiao M, Li Q, Ren W, Li S, Gao M, Pang Y (2022). Increased tuberculosis incidence due to immunotherapy based on PD-1 and PD-L1 blockade: a systematic review and meta-analysis. Frontiers in Immunology.

[bib71] Lu LL, Smith MT, Yu KKQ, Luedemann C, Suscovich TJ, Grace PS, Cain A, Yu WH, McKitrick TR, Lauffenburger D, Cummings RD, Mayanja-Kizza H, Hawn TR, Boom WH, Stein CM, Fortune SM, Seshadri C, Alter G (2019). IFN-γ-independent immune markers of *Mycobacterium tuberculosis* exposure. Nature Medicine.

[bib72] Luo Y, Huang CC, Howard NC, Wang X, Liu Q, Li X, Zhu J, Amariuta T, Asgari S, Ishigaki K, Calderon R, Raman S, Ramnarine AK, Mayfield JA, Moody DB, Lecca L, Fortune SM, Murray MB, Raychaudhuri S (2024). Paired analysis of host and pathogen genomes identifies determinants of human tuberculosis. Nature Communications.

[bib73] Mangtani P, Abubakar I, Ariti C, Beynon R, Pimpin L, Fine PEM, Rodrigues LC, Smith PG, Lipman M, Whiting PF, Sterne JA (2014). Protection by BCG vaccine against tuberculosis: a systematic review of randomized controlled trials. Clinical Infectious Diseases.

[bib74] Mann M, Kumar C, Zeng WF, Strauss MT (2021). Artificial intelligence for proteomics and biomarker discovery. Cell Systems.

[bib75] Marakalala MJ, Raju RM, Sharma K, Zhang YJ, Eugenin EA, Prideaux B, Daudelin IB, Chen PY, Booty MG, Kim JH, Eum SY, Via LE, Behar SM, Barry CE, Mann M, Dartois V, Rubin EJ (2016). Inflammatory signaling in human tuberculosis granulomas is spatially organized. Nature Medicine.

[bib76] McCaffrey EF, Donato M, Keren L, Chen Z, Delmastro A, Fitzpatrick MB, Gupta S, Greenwald NF, Baranski A, Graf W, Kumar R, Bosse M, Fullaway CC, Ramdial PK, Forgó E, Jojic V, Van Valen D, Mehra S, Khader SA, Bendall SC, van de Rijn M, Kalman D, Kaushal D, Hunter RL, Banaei N, Steyn AJC, Khatri P, Angelo M (2022). The immunoregulatory landscape of human tuberculosis granulomas. Nature Immunology.

[bib77] Moorlag SJCFM, Arts RJW, van Crevel R, Netea MG (2019). Non-specific effects of BCG vaccine on viral infections. Clinical Microbiology and Infection.

[bib78] Nathan C (2023). *Mycobacterium tuberculosis* as teacher. Nature Microbiology.

[bib79] O’Garra A, Redford PS, McNab FW, Bloom CI, Wilkinson RJ, Berry MPR (2013). The immune response in tuberculosis. Annual Review of Immunology.

[bib80] Olive AJ, Sassetti CM (2018). Tolerating the unwelcome guest; how the host withstands persistent *Mycobacterium tuberculosis*. Frontiers in Immunology.

[bib81] Ong CWM, Elkington PT, Brilha S, Ugarte-Gil C, Tome-Esteban MT, Tezera LB, Pabisiak PJ, Moores RC, Sathyamoorthy T, Patel V, Gilman RH, Porter JC, Friedland JS (2015). Neutrophil-Derived MMP-8 Drives AMPK-dependent matrix destruction in human pulmonary tuberculosis. PLOS Pathogens.

[bib82] Pagán AJ, Ramakrishnan L (2018). The formation and function of granulomas. Annual Review of Immunology.

[bib83] Palla G, Fischer DS, Regev A, Theis FJ (2022). Spatial components of molecular tissue biology. Nature Biotechnology.

[bib84] Patterson B, Dinkele R, Gessner S, Koch A, Hoosen Z, January V, Leonard B, McKerry A, Seldon R, Vazi A, Hermans S, Cobelens F, Warner DF, Wood R (2024). Aerosolization of viable *Mycobacterium tuberculosis* bacilli by tuberculosis clinic attendees independent of sputum-Xpert Ultra status. PNAS.

[bib85] Pittet LF, Messina NL, Orsini F, Moore CL, Abruzzo V, Barry S, Bonnici R, Bonten M, Campbell J, Croda J, Dalcolmo M, Gardiner K, Gell G, Germano S, Gomes-Silva A, Goodall C, Gwee A, Jamieson T, Jardim B, Kollmann TR, Lacerda MVG, Lee KJ, Lucas M, Lynn DJ, Manning L, Marshall HS, McDonald E, Munns CF, Nicholson S, O’Connell A, de Oliveira RD, Perlen S, Perrett KP, Prat-Aymerich C, Richmond PC, Rodriguez-Baño J, Dos Santos G, da Silva PV, Teo JW, Villanueva P, Warris A, Wood NJ, Davidson A, Curtis N, BRACE Trial Consortium Group (2023). Randomized trial of BCG vaccine to protect against Covid-19 in health care workers. The New England Journal of Medicine.

[bib86] Proulx MK, Wiggins CD, Reames CJ, Wu C, Kiritsy MC, Xu P, Gallant JC, Grace PS, Fenderson BA, Smith CM, Lindestam Arlehamn CS, Alter G, Lauffenburger DA, Sassetti CM (2025). Noncanonical T cell responses are associated with protection from tuberculosis in mice and humans. The Journal of Experimental Medicine.

[bib87] Reid M, Agbassi YJP, Arinaminpathy N, Bercasio A, Bhargava A, Bhargava M, Bloom A, Cattamanchi A, Chaisson R, Chin D, Churchyard G, Cox H, Denkinger CM, Ditiu L, Dowdy D, Dybul M, Fauci A, Fedaku E, Gidado M, Harrington M, Hauser J, Heitkamp P, Herbert N, Herna Sari A, Hopewell P, Kendall E, Khan A, Kim A, Koek I, Kondratyuk S, Krishnan N, Ku C-C, Lessem E, McConnell EV, Nahid P, Oliver M, Pai M, Raviglione M, Ryckman T, Schäferhoff M, Silva S, Small P, Stallworthy G, Temesgen Z, van Weezenbeek K, Vassall A, Velásquez GE, Venkatesan N, Yamey G, Zimmerman A, Jamison D, Swaminathan S, Goosby E (2023). Scientific advances and the end of tuberculosis: a report from the Lancet Commission on Tuberculosis. Lancet.

[bib88] Rieckmann A, Villumsen M, Sørup S, Haugaard LK, Ravn H, Roth A, Baker JL, Benn CS, Aaby P (2017). Vaccinations against smallpox and tuberculosis are associated with better long-term survival: a Danish case-cohort study 1971-2010. International Journal of Epidemiology.

[bib89] Rosas Mejia O, Gloag ES, Li J, Ruane-Foster M, Claeys TA, Farkas D, Wang S-H, Farkas L, Xin G, Robinson RT (2022). Mice infected with *Mycobacterium tuberculosis* are resistant to acute disease caused by secondary infection with SARS-CoV-2. PLOS Pathogens.

[bib90] Russell DG (2011). *Mycobacterium tuberculosis* and the intimate discourse of a chronic infection. Immunological Reviews.

[bib91] Ryckman TS, Dowdy DW, Kendall EA (2022). Infectious and clinical tuberculosis trajectories: Bayesian modeling with case finding implications. PNAS.

[bib92] Sawyer AJ, Patrick E, Edwards J, Wilmott JS, Fielder T, Yang Q, Barber DL, Ernst JD, Britton WJ, Palendira U, Chen X, Feng CG (2023). Spatial mapping reveals granuloma diversity and histopathological superstructure in human tuberculosis. The Journal of Experimental Medicine.

[bib93] Schmidt AC, Fairlie L, Hellström E, Luabeya Kany Kany A, Middelkoop K, Naidoo K, Nair G, Gela A, Nemes E, Scriba TJ, Cinar A, Frahm N, Mogg R, Kaufman D, Dunne MW, Hatherill M, BCG REVAX Study Team (2025). BCG revaccination for the prevention of *Mycobacterium tuberculosis* infection. The New England Journal of Medicine.

[bib94] Schurz H, Naranbhai V, Yates TA, Gilchrist JJ, Parks T, Dodd PJ, Möller M, Hoal EG, Morris AP, Hill AVS, International Tuberculosis Host Genetics Consortium (2024). Multi-ancestry meta-analysis of host genetic susceptibility to tuberculosis identifies shared genetic architecture. eLife.

[bib95] Simmons JD, Dill-McFarland KA, Stein CM, Van PT, Chihota V, Ntshiqa T, Maenetje P, Peterson GJ, Benchek P, Nsereko M, Velen K, Fielding KL, Grant AD, Gottardo R, Mayanja-Kizza H, Wallis RS, Churchyard G, Boom WH, Hawn TR (2022). Monocyte transcriptional responses to *Mycobacterium tuberculosis* associate with resistance to tuberculin skin test and interferon gamma release assay conversion. mSphere.

[bib96] Smith CM, Baker RE, Proulx MK, Mishra BB, Long JE, Park SW, Lee H-N, Kiritsy MC, Bellerose MM, Olive AJ, Murphy KC, Papavinasasundaram K, Boehm FJ, Reames CJ, Meade RK, Hampton BK, Linnertz CL, Shaw GD, Hock P, Bell TA, Ehrt S, Schnappinger D, Pardo-Manuel de Villena F, Ferris MT, Ioerger TR, Sassetti CM (2022). Host-pathogen genetic interactions underlie tuberculosis susceptibility in genetically diverse mice. eLife.

[bib97] Stensballe LG, Nante E, Jensen IP, Kofoed P-E, Poulsen A, Jensen H, Newport M, Marchant A, Aaby P (2005). Acute lower respiratory tract infections and respiratory syncytial virus in infants in Guinea-Bissau: a beneficial effect of BCG vaccination for girls community based case-control study. Vaccine.

[bib98] Sun L, Guo W, Guo L, Chen X, Zhou H, Yan S, Zhao G, Bao H, Wu X, Shao Y, Ying J, Lin L (2024a). Molecular landscape and multi-omic measurements of heterogeneity in fetal adenocarcinoma of the lung. NPJ Precision Oncology.

[bib99] Sun M, Phan JM, Kieswetter NS, Huang H, Yu KKQ, Smith MT, Liu YE, Wang C, Gupta S, Obermoser G, Maecker HT, Krishnan A, Suresh S, Gupta N, Rieck M, Acs P, Ghanizada M, Chiou S-H, Khatri P, Boom WH, Hawn TR, Stein CM, Mayanja-Kizza H, Davis MM, Seshadri C (2024b). Specific CD4^+^ T cell phenotypes associate with bacterial control in people who “resist” infection with *Mycobacterium tuberculosis*. Nature Immunology.

[bib100] Swanson RV, Gupta A, Foreman TW, Lu L, Choreno-Parra JA, Mbandi SK, Rosa BA, Akter S, Das S, Ahmed M, Garcia-Hernandez MDLL, Singh DK, Esaulova E, Artyomov MN, Gommerman J, Mehra S, Zuniga J, Mitreva M, Scriba TJ, Rangel-Moreno J, Kaushal D, Khader SA (2023). Antigen-specific B cells direct T follicular-like helper cells into lymphoid follicles to mediate *Mycobacterium tuberculosis* control. Nature Immunology.

[bib101] Tait DR, Hatherill M, Van Der Meeren O, Ginsberg AM, Van Brakel E, Salaun B, Scriba TJ, Akite EJ, Ayles HM, Bollaerts A, Demoitié M-A, Diacon A, Evans TG, Gillard P, Hellström E, Innes JC, Lempicki M, Malahleha M, Martinson N, Mesia Vela D, Muyoyeta M, Nduba V, Pascal TG, Tameris M, Thienemann F, Wilkinson RJ, Roman F (2019). Final analysis of a trial of M72/AS01_E_ vaccine to prevent tuberculosis. The New England Journal of Medicine.

[bib102] Tarazona S, Balzano-Nogueira L, Gómez-Cabrero D, Schmidt A, Imhof A, Hankemeier T, Tegnér J, Westerhuis JA, Conesa A (2020). Harmonization of quality metrics and power calculation in multi-omic studies. Nature Communications.

[bib103] Tarazona S, Arzalluz-Luque A, Conesa A (2021). Undisclosed, unmet and neglected challenges in multi-omics studies. Nature Computational Science.

[bib104] Tezera LB, Bielecka MK, Ogongo P, Walker NF, Ellis M, Garay-Baquero DJ, Thomas K, Reichmann MT, Johnston DA, Wilkinson KA, Ahmed M, Jogai S, Jayasinghe SN, Wilkinson RJ, Mansour S, Thomas GJ, Ottensmeier CH, Leslie A, Elkington PT (2020a). Anti-PD-1 immunotherapy leads to tuberculosis reactivation via dysregulation of TNF-α. eLife.

[bib105] Tezera LB, Mansour S, Elkington P (2020b). Reconsidering the optimal immune response to *Mycobacterium tuberculosis*. American Journal of Respiratory and Critical Care Medicine.

[bib106] Toussaint PA, Leiser F, Thiebes S, Schlesner M, Brors B, Sunyaev A (2023). Explainable artificial intelligence for omics data: a systematic mapping study. Briefings in Bioinformatics.

[bib107] Trajman A, Campbell JR, Kunor T, Ruslami R, Amanullah F, Behr MA, Menzies D (2025). Tuberculosis. The Lancet.

[bib108] Trunz BB, Fine P, Dye C (2006). Effect of BCG vaccination on childhood tuberculous meningitis and miliary tuberculosis worldwide: a meta-analysis and assessment of cost-effectiveness. Lancet.

[bib109] Vallini L, Zampieri C, Shoaee MJ, Bortolini E, Marciani G, Aneli S, Pievani T, Benazzi S, Barausse A, Mezzavilla M, Petraglia MD, Pagani L (2024). The Persian plateau served as hub for *Homo sapiens* after the main out of Africa dispersal. Nature Communications.

[bib110] Verrall AJ, Schneider M, Alisjahbana B, Apriani L, van Laarhoven A, Koeken VACM, van Dorp S, Diadani E, Utama F, Hannaway RF, Indrati A, Netea MG, Sharples K, Hill PC, Ussher JE, van Crevel R (2020). Early clearance of *Mycobacterium tuberculosis* is associated with increased innate immune responses. The Journal of Infectious Diseases.

[bib111] Winchell CG, Nyquist SK, Chao MC, Maiello P, Myers AJ, Hopkins F, Chase M, Gideon HP, Patel KV, Bromley JD, Simonson AW, Floyd-O’Sullivan R, Wadsworth M, Rosenberg JM, Uddin R, Hughes T, Kelly RJ, Griffo J, Tomko J, Klein E, Berger B, Scanga CA, Mattila J, Fortune SM, Shalek AK, Lin PL, Flynn JL (2023). CD8+ lymphocytes are critical for early control of tuberculosis in macaques. The Journal of Experimental Medicine.

[bib112] Yenew B, Ghodousi A, Diriba G, Tesfaye E, Cabibbe AM, Amare M, Moga S, Alemu A, Dagne B, Sinshaw W, Mollalign H, Meaza A, Tadesse M, Gamtesa DF, Abebaw Y, Seid G, Zerihun B, Getu M, Chiacchiaretta M, Gaudin C, Marceau M, Didelot X, Tolera G, Abdella S, Kebede A, Getahun M, Mehammed Z, Supply P, Cirillo DM (2023). A smooth tubercle bacillus from Ethiopia phylogenetically close to the *Mycobacterium tuberculosis* complex. Nature Communications.

[bib113] Yoder MA, Lamichhane G, Bishai WR (2004). Cavitary pulmonary tuberculosis: The Holey Grail of disease transmission. Current Science.

[bib114] Young D (2009). Animal models of tuberculosis. European Journal of Immunology.

[bib115] Zheng Y, Liu Y, Yang J, Dong L, Zhang R, Tian S, Yu Y, Ren L, Hou W, Zhu F, Mai Y, Han J, Zhang L, Jiang H, Lin L, Lou J, Li R, Lin J, Liu H, Kong Z, Wang D, Dai F, Bao D, Cao Z, Chen Q, Chen Q, Chen X, Gao Y, Jiang H, Li B, Li B, Li J, Liu R, Qing T, Shang E, Shang J, Sun S, Wang H, Wang X, Zhang N, Zhang P, Zhang R, Zhu S, Scherer A, Wang J, Wang J, Huo Y, Liu G, Cao C, Shao L, Xu J, Hong H, Xiao W, Liang X, Lu D, Jin L, Tong W, Ding C, Li J, Fang X, Shi L (2024). Multi-omics data integration using ratio-based quantitative profiling with Quartet reference materials. Nature Biotechnology.

[bib116] Zhu J, He Z, Liang D, Yu X, Qiu K, Wu J (2022). Pulmonary tuberculosis associated with immune checkpoint inhibitors: a pharmacovigilance study. Thorax.

